# Why the estrous cycle matters for neuroscience

**DOI:** 10.1186/s13293-022-00466-8

**Published:** 2022-10-28

**Authors:** Devin Rocks, Heining Cham, Marija Kundakovic

**Affiliations:** 1 Department of Biological Sciences, Fordham University, 441 E. Fordham Road, Larkin Hall 160, Bronx, NY 10458 USA; 2 Department of Psychology, Fordham University, 441 E. Fordham Road, Dealy Hall 226G, Bronx, NY 10458 USA

## Abstract

**Background:**

Ovarian hormone fluctuations over the rodent estrous cycle and the human menstrual cycle are known to significantly impact brain physiology and disease risk, yet this variable is largely ignored in preclinical neuroscience research, clinical studies, and psychiatric practice.

**Methods:**

To assess the importance of the estrous cycle information for the analysis of sex differences in neuroscience research, we re-analyzed our previously published data with or without the estrous cycle information, giving a side-by-side comparison of the analyses of behavior, brain structure, gene expression, and 3D genome organization in female and male mice. We also examined and compared the variance of female and male groups across all neurobehavioral measures.

**Results:**

We show that accounting for the estrous cycle significantly increases the resolution of the neuroscience studies and allows for: (a) identification of masked sex differences; (b) mechanistic insight(s) into the identified sex differences, across different neurobehavioral outcomes, from behavior to molecular phenotypes. We confirm previous findings that female data from either mixed- or staged-female groups are, on average, not more variable than that of males. However, we show that female variability is not, at all, predictive of whether the estrous cycle plays an important role in regulating the outcome of interest.

**Conclusions:**

We argue that “bringing back” the estrous cycle variable to the main stage is important in order to enhance the resolution and quality of the data, to advance the health of women and other menstruators, and to make research more gender-inclusive. We strongly encourage the neuroscience community to incorporate the estrous cycle information in their study design and data analysis, whenever possible, and we debunk some myths that tend to de-emphasize the importance and discourage the inclusion of this critically important biological variable.

**Highlights**

- Ovarian hormone fluctuation impacts brain physiology and is a major psychiatric risk factor, yet this variable has been overlooked in neuroscience research and psychiatric practice.
- From rodent behavior to gene regulation, accounting for the estrous cycle increases the resolution of the neuroscience data, allowing identification and mechanistic insight(s) into sex differences.
- Female variability does not equal (and is not predictive of) the estrous cycle effect and should not be used as a proxy for the effects of ovarian hormones on the outcome of interest.
- Neuroscience researchers are advised to incorporate the estrous cycle information in their studies to foster more equitable, female- and gender-inclusive research.
- Studies of the ovarian cycle are especially important for improving women’s mental health.

## Background

Ovarian hormone fluctuation shapes brain physiology [1–7] and represents a major psychiatric risk factor in humans [8–14], yet this variable is largely ignored in preclinical neuroscience research, clinical studies, and psychiatric practice [8, 15–17]. The psychiatric risk in women is elevated throughout the reproductive period and is directly linked to hormonal changes [17]. With the first menarche and the onset of hormonal cycles, risk for depression in girls increases two times compared to that of boys [13]. Up to 20% of pregnant people develop depression following the sharp decrease in circulating sex hormone levels postpartum [11, 12]. 5–8% of women suffer from premenstrual dysphoric disorder (PMDD) due to an increased sensitivity to physiological hormone fluctuations [10]. More than 50% of patients with depression, bipolar, and anxiety disorders also report worsening of their symptoms premenstrually [9, 18]. During menopausal transition, with the most erratic hormone changes and severe estrogen drop, the risk for depression increases 2–5 times [14]. With all this in mind, how is it possible that neuroscience tends to ignore the effect of ovarian hormone fluctuation on the female brain and behavior?

The answer goes back to decades of preclinical neuroscience research that focused on the male brain. Approximately, for every 5.5 studied male animals, only one female animal was examined in neurosciences, as reported in 2011 [19]. The main reason for excluding females was said to be a higher female variability due to the above-mentioned effects of reproductive hormone cycles on the brain and behavioral outcomes. Researchers claimed that including females would require including additional experimental groups, more expensive experiments, and more complicated data analyses [20]. From our point of view, it seems unacceptable that one sex (and approximately 50% of the population) can be dismissed and understudied because of its “complicated biology”. Notably, this erroneous practice led to our limited understanding of the female brain and its intrinsic hormone-driven plasticity.

More recently, there have been important calls for including females in biomedical research as a necessary step for a more equitable research practice and to enable an understanding of sex-specific brain physiology and disease risk [21–23]. Unfortunately, while well-intentioned, the voices that promoted inclusion of females, also de-emphasized the importance of the estrous cycle as a variable. Two major meta-analyses performed in mice [22] and in rats [21] provided the evidence that, in general, females are not more variable than males across neuroscience-related outcomes. This led to a trend that some researchers called “liberation of female animals” [22], implying that because females are similar to males in terms of variability, the estrous cycle can be dismissed, and that therefore females “deserve to be studied”. And, this perceived need to justify the inclusion of females by rejecting female complexity, rather than by acknowledging a simple need for inclusivity, is still promoted to this day [23–26].

Responding to calls for female inclusion, in 2016, the NIH mandated the use of both sexes in all experiments, under the policy known as Sex as a Biological Variable (SABV) [27]. Preliminary studies have shown limited success of this approach; more studies started reporting sex and including both males and females, but very few studies included sex as a variable in their data analysis [28–30]. It also became clear that some researchers would promise using females in their grant proposals but would never deliver on their promise in their publications; there has been no system in place to keep researchers accountable, either by funding agencies or by the scientific journals. The percentage of female-only studies was kept steadily at less than 5% [30] and de-emphasizing of the estrous cycle in the studies of both sexes led to further marginalization of the importance of natural hormonal shifts on female brain health, locking this research into a small niche field of neuroendocrinology or women’s health research, although ovarian hormonal shifts affect more than one-fourth of the population at any given time [2].

Fast-forward to the past year or so, another acute problem in sex difference research has been called out. Voices have been raised that sex is a non-binary, multilayered, and context-dependent biological variable [31]; that sex differences may have been misreported due to improper design and data analyses [32]; and that, as such, SABV may hurt rather than help precision medicine initiatives [33]. In fact, rather than thinking about sex as a single variable, we may want to look at sex as a composite variable whose components, such as hormonal status or sex chromosome complement, can explain the sex-based variation better than sex, while at the same time allowing for more gender-inclusive research practices.

**Fig. 1:**
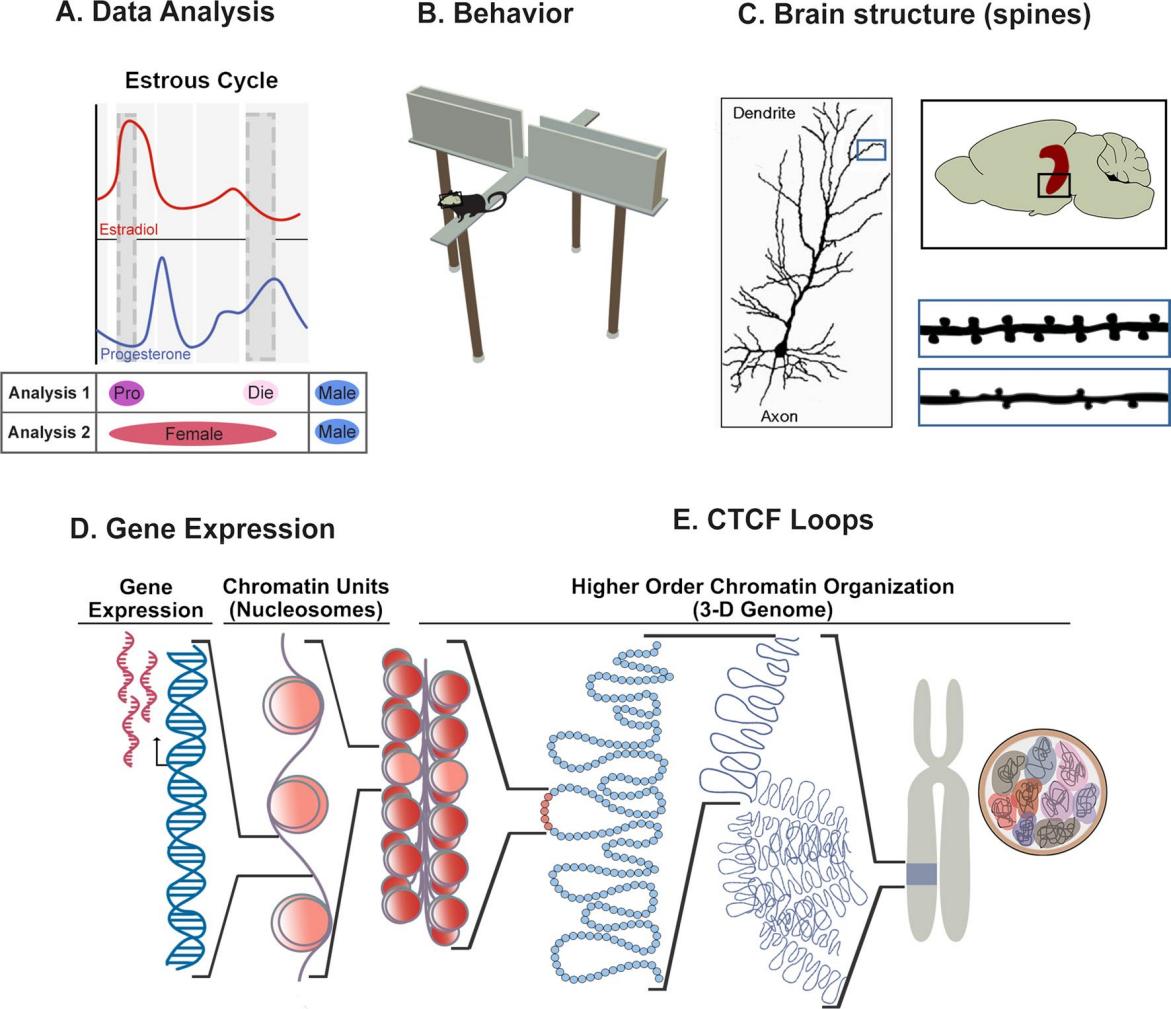
Study approach across neurobehavioral outcomes. **A** We performed two types of analyses. **Analysis 1** took the estrous cycle information into account, comparing proestrus females (high estradiol–low progesterone), diestrus females (low estradiol–high progesterone), and males. **Analysis 2** compared merged females (proestrus + diestrus) with males. The above diagram shows physiological fluctuations of ovarian hormones, estradiol and progesterone, over the rodent estrous cycle. **B** The first neurobehavioral outcomes analyzed included anxiety-related behavioral tests in mice such as the elevated plus maze (depicted). **C** The second level of analysis included spine density of dendrites located on neurons in the ventral hippocampal region, critical for control of anxiety-related behaviors. **D** We then analyzed molecular phenotypes including gene expression in the ventral hippocampus. **E** Finally, we assessed gene regulatory mechanisms in ventral hippocampal neurons including features of the higher-order chromatin organization such as CTCF loops. Pro (purple), proestrus; Die (light pink), diestrus; Female (red), mixed females; Male (blue), males.

Our paper addresses all above-mentioned points of view. We take a practical approach, and by re-analyzing our previously published data [5, 34] with or without the estrous cycle information (Fig. 1A), we show that accounting for the estrous cycle, as an exemplary sex-specific factor, makes the data more interpretable and increases our ability to discover and explain sex differences. We also challenge the narrative surrounding the findings that females are, in general, not more variable than males. While our data are in agreement with this finding, we show that female variability is not, at all, predictive of whether the estrous cycle is playing an important role in regulating an outcome of interest. Moreover, and most importantly, we argue that the question is not whether females should be studied or not, the question is only how best to study females. We strongly encourage the neuroscience community to incorporate the estrous cycle information in their study design and data analyses, whenever possible, in order to enhance the resolution and quality of the data and to advance the so-much-neglected health of women and other menstruating individuals, which is critically shaped by an individual’s hormonal status.

## Methods

**Fig. 2:**
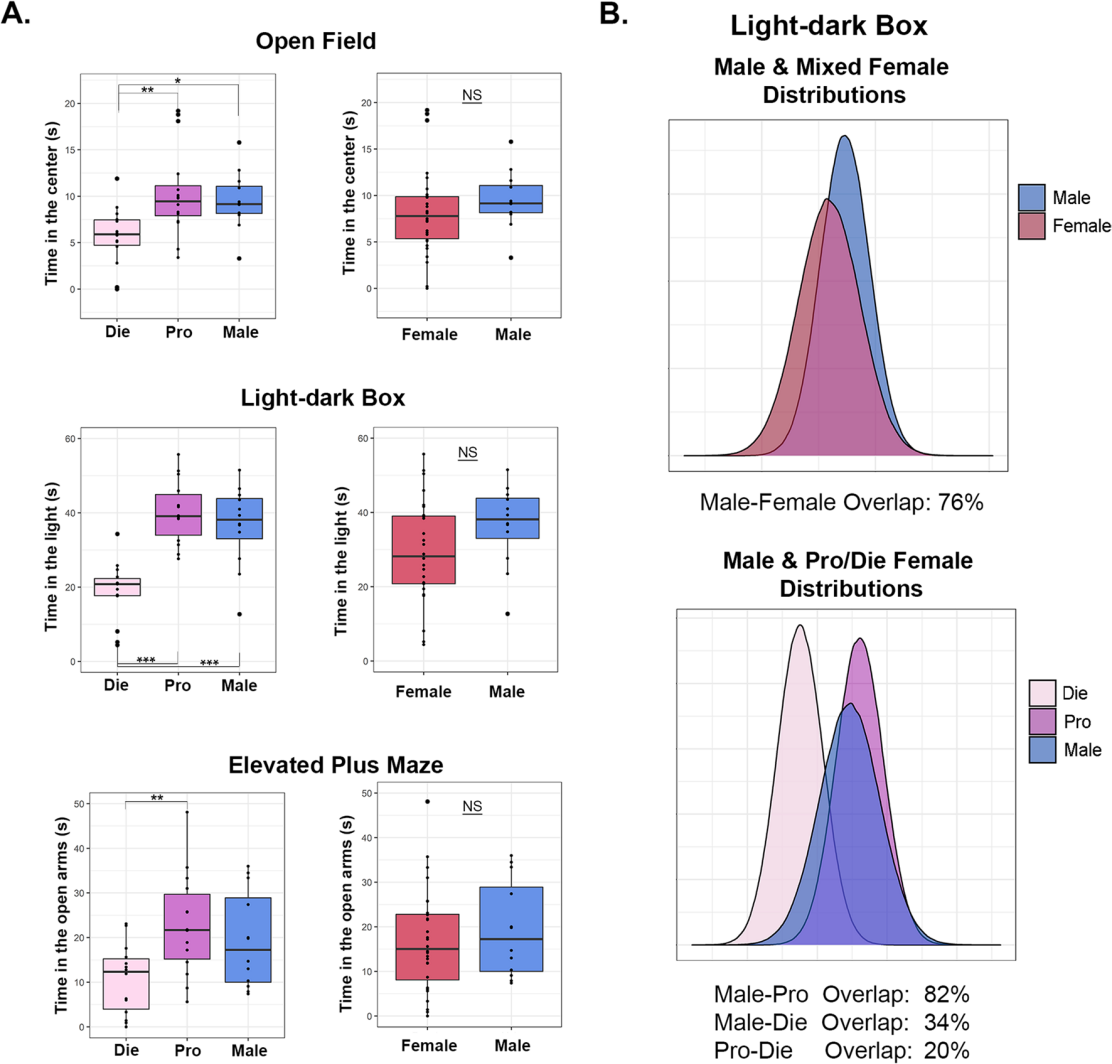
Estrous cycle information is required to detect sex differences in anxiety-related behavior. **A** Behavioral data are shown for the open field, light–dark box, and elevated plus maze tests. On the left, we reproduced our previously published data in which females are separated into proestrus and diestrus phases and compared to males [5]. On the right, we performed the re-analysis of the data by comparing the merged female group (proestrus + diestrus) to males. **B** Density distribution plots depict the normal distributions of the light–dark box data for merged female groups compared to males (top) and males compared to females separated by the estrous cycle stage (bottom). The degree of overlap between the distributions is given below each plot. Box plots (box, 1st–3rd quartile; horizontal line, median; whiskers, 1.5 × IQR); *NS* not significant; *P < 0.05; **P < 0.01; ***P < 0.001; one-way ANOVA with the Tukey’s post hoc test (left); Welch two-sample T-test (right). Die (light pink), diestrus; Pro (purple), proestrus; Female (red), mixed females; Male (blue), males. *n* = 12–16 animals/group

**Fig. 3:**
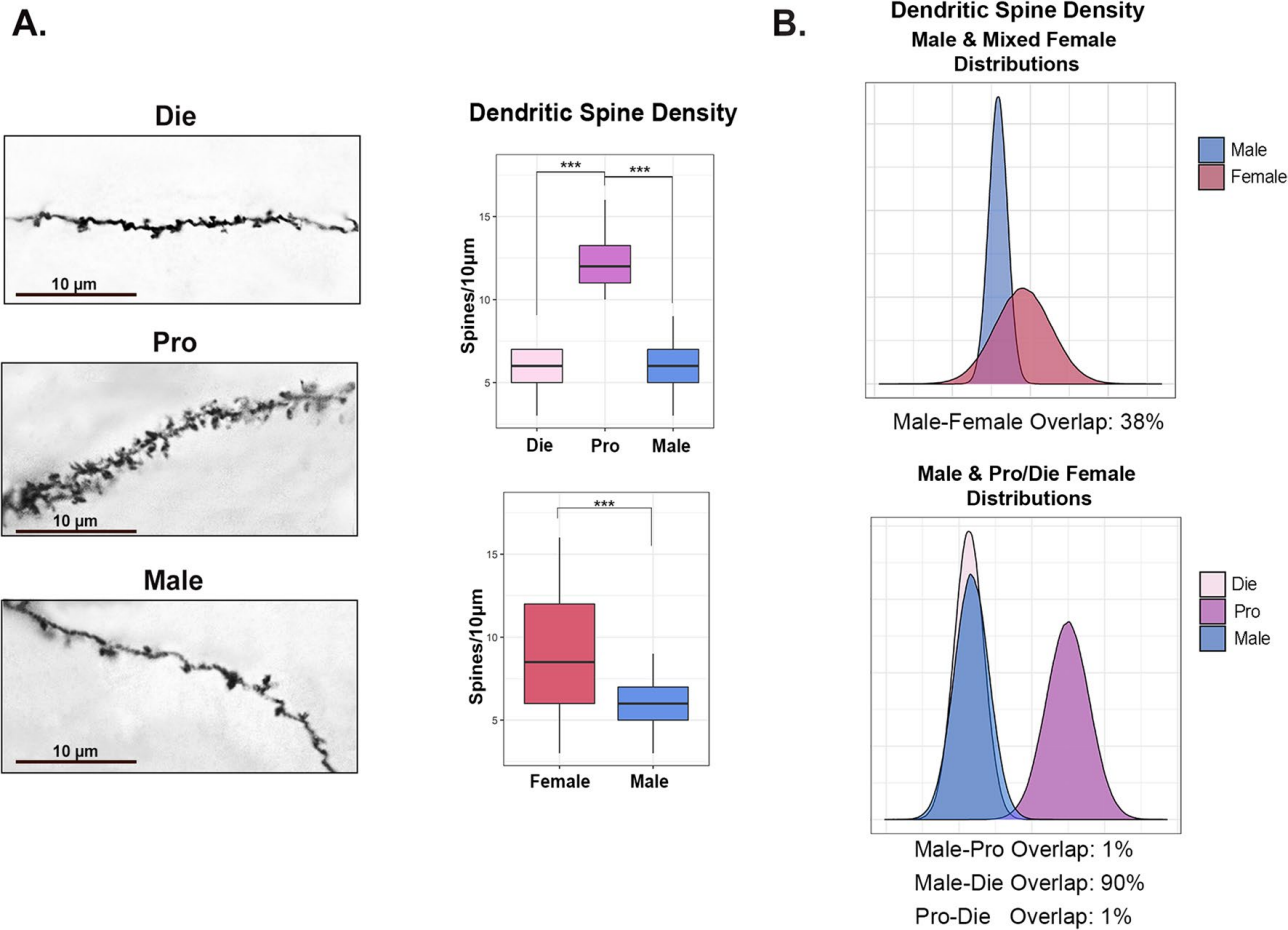
Sex differences in ventral hippocampal dendritic spine density are estrous stage-dependent. **A** Representative photomicrographs of dendritic spine density (Golgi staining, *n* = 5 animals/group; scale bar: 10 μm) in the ventral hippocampus of diestrus females, proestrus females, and males are shown (left; adapted from Jaric et al. [5]) along with the quantification of the data (right) for the three groups (top; reproduced from [5]) as well as the comparison between males and merged females (bottom). **B** Density distribution plots representing normal distributions of dendritic spine density for merged females compared to males (top) and males compared to proestrus and diestrus females (bottom). The degree of overlap between the distributions is given below each plot. Box plots (box, 1st–3rd quartile; horizontal line, median; whiskers, 1.5 × IQR); ***P < 0.001; one-way ANOVA with the Tukey’s post hoc test (top); Welch Two-sample *T*-test (bottom). Die (light pink), diestrus; Pro (purple), proestrus; Female (red), mixed females; Male (blue), males

**Fig. 4:**
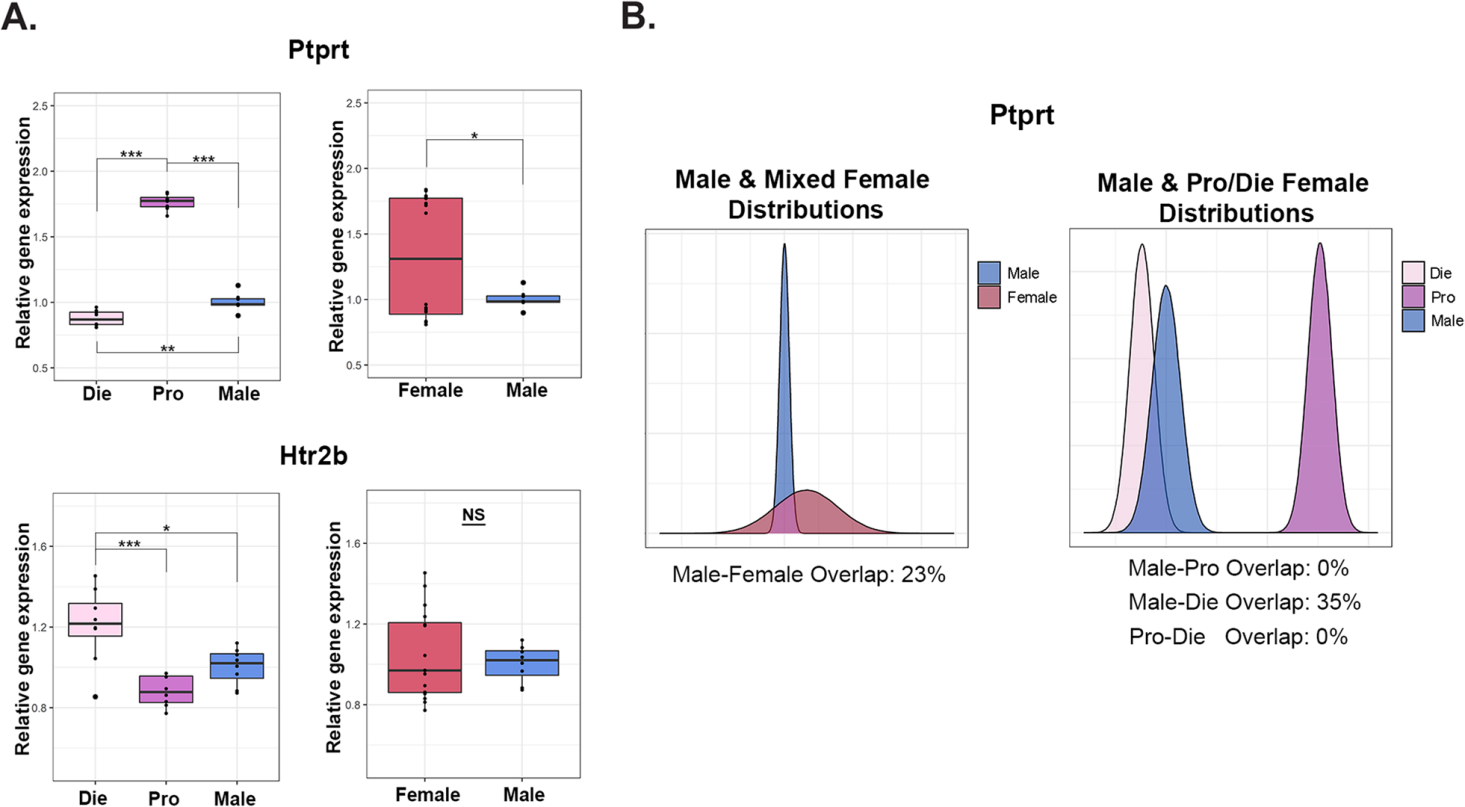
Sex differences in gene expression depend on the estrous cycle. **A** *Ptprt* (top) and *Htr2b* (bottom) mRNA levels in the ventral hippocampus of diestrus females, proestrus females, and males (left; reproduced from [5]) as well as males and merged-females (right). **B** Density distribution plots of *Ptprt* gene expression for merged females compared to males (left) and males compared to females separated by estrous cycle stage (right). The degree of overlap between the distributions is given below each plot. Box plots (box, 1st–3rd quartile; horizontal line, median; whiskers, 1.5 × IQR); NS- not significant; *P < 0.05; **P < 0.01; ***P < 0.001; one-way ANOVA with the Tukey’s post hoc test (left); Welch two-sample T-test (right). Die (light pink), diestrus; Pro (purple), proestrus; Female (red), mixed females; Male (blue), males. *n* = 8 animals/group

*Data* To assess the importance of the estrous cycle information for the analysis of sex differences in neuroscience research, we reanalyzed our previously published data [5, 34] generated in young adult (8–11 weeks old) male and female C57BL/6J mice. In females, we tracked the estrous cycle daily, for the duration of three cycles [5], and included mice in two extreme phases of the estrous cycle: proestrus (high estradiol–low progesterone) and early diestrus (low estradiol–high progesterone) that mimic human follicular and luteal phases, respectively (Fig. 1A). To illustrate the effects of estrous cycle classification on the statistical outcomes and data interpretation, we give a side-by-side comparison of analyses comparing males, proestrus females, and diestrus females (Analysis 1) to analyses which include males and mixed-females (Analysis 2, Fig. 1A) across four different neurobehavioral outcomes including behavior (Fig. 1B), brain structure (Fig. 1C), gene expression (Fig. 1D), and 3D chromatin organization (Fig. 1E). A total of six datasets were analyzed statistically: results of three anxiety-related behavioral tests (*n* = 12–16 animals/group; Fig. 2); ventral hippocampal dendritic spine density data (*n* = 200 images from *n* = 5 animals/group; Fig. 3); and gene expression data for two genes assessed with qRT-PCR (*n* = 8 animals/group, Fig. 4).

*Data re-analysis* For Analysis 1, data were re-analyzed using one-way ANOVA with Tukey’s post hoc test, as previously published [5]. For Analysis 2, data were merged from proestrus females and diestrus females to form one female group, and the data from females and males were compared using a Welch two-sample *T*-test (Fig. 1A). All statistical analyses and plots of these data were generated using R software. The degree of overlap in the distribution plots was determined using the SexDifference.org web tool [35].

**Fig. 5:**
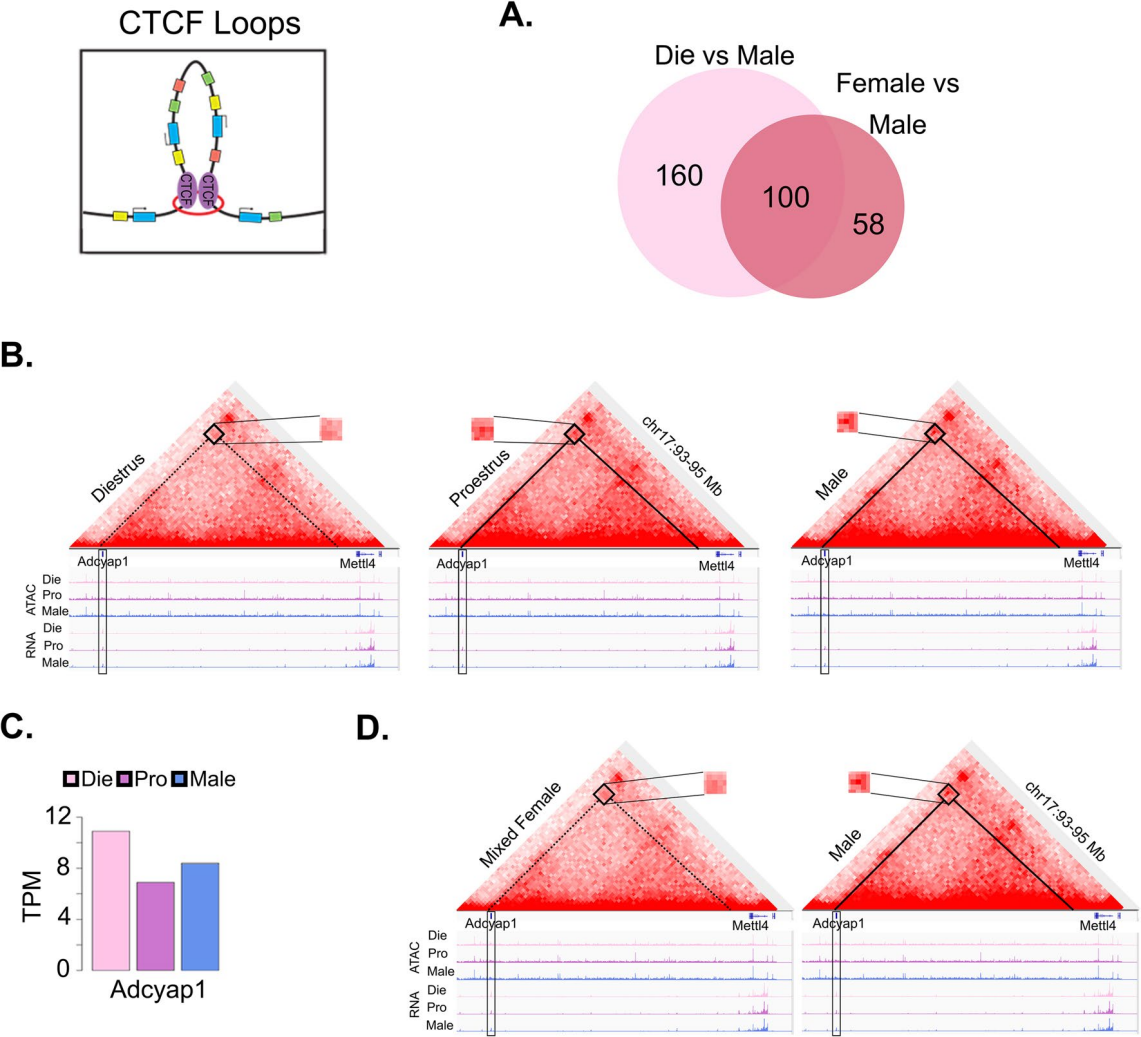
Estrous cycle information enhances the ability to identify sex-specific CTCF loops in neuronal chromatin. CTCF loops (top left) allow long-range interactions in the genome of relevance to gene regulation. **A** Venn diagram (top right) depicts differential CTCF loops called in the diestrus–male comparison and differential loops called in the mixed-female to male comparison. **B** A HiC heatmap of a 2 Mb loop connecting the *Adcyap1* locus with a region upstream of *Mettl4* is shown, with a higher signal in males and proestrus females (solid line) compared to diestrus females (dashed line). **C** Differential CTCF loops correspond to differences in *Adcyap1* gene expression. **D** When comparing males to mixed females, the averaged signal from females for this loop is weaker than that of males. The corresponding ATAC-seq (chromatin accessibility) and RNA-seq (gene expression) tracks are shown below the Hi-C data. Die (light pink), diestrus; Pro (purple), proestrus; Male (blue), males. All data are derived from 3 biological replicates (*n* = 6 animals) per group (adapted from [34])

*CTCF loops* The analysis of differential CTCF loops, identified using the Hi-C assay, is described in previous publication of this data [34]. Data were generated from fluorescence-activated nuclei sorting (FANS)-purified neuronal (NeuN +) nuclei isolated from the ventral hippocampus of proestrus females, diestrus females, and males (*n* = 3 biological replicates/group; tissue pooled from 2 animals/replicate). Differential CTCF loops were assessed between diestrus females and males as well as between merged females and males (Fig. 5).

**Fig. 6:**
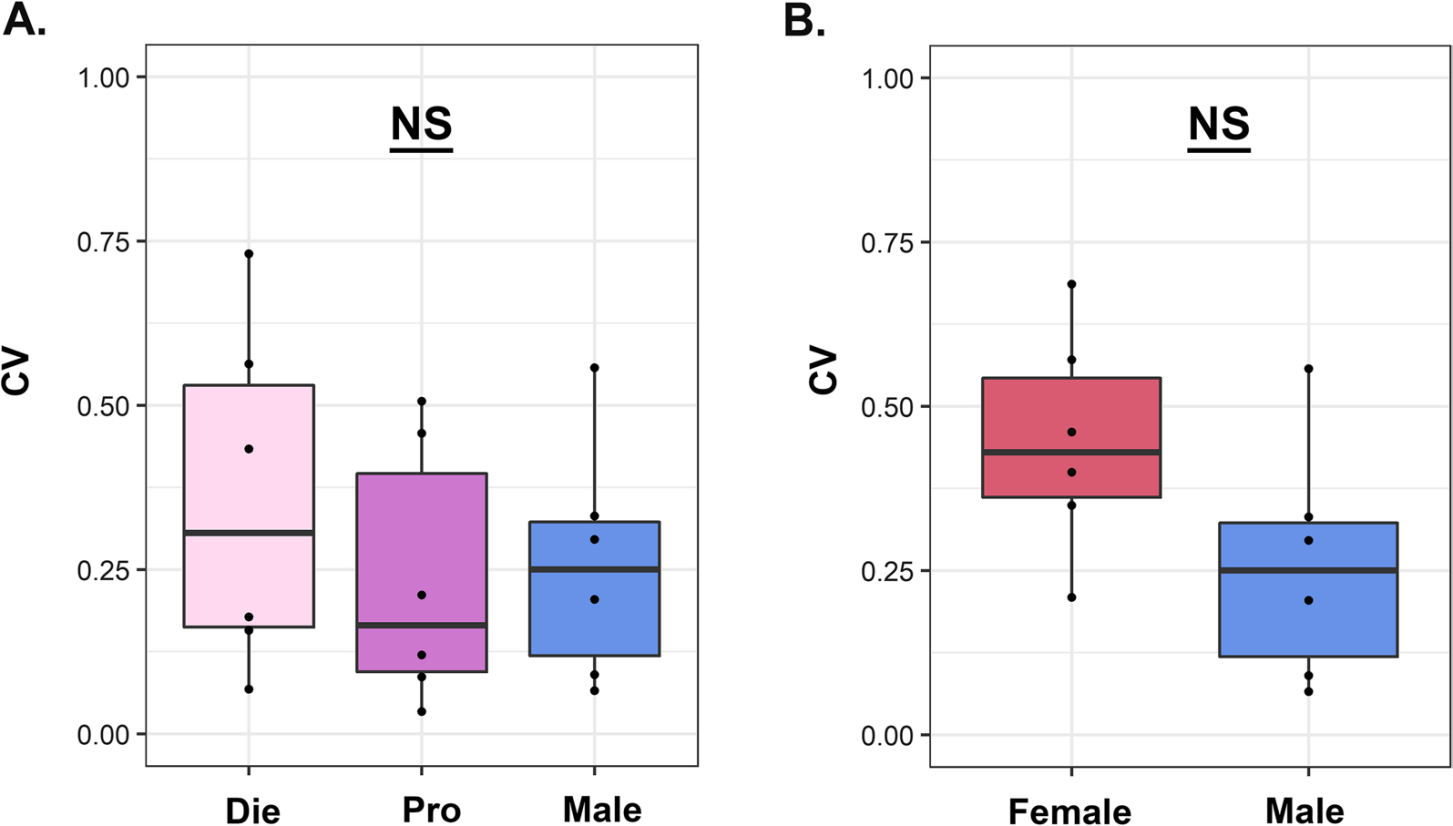
Females are not more variable than males across the analyzed datasets. Coefficient of variation (CV) comparison is shown across neurobehavioral measures in males and females: **A** Taking into account the estrous cycle; or **B** in merged females vs. males. NS, not significant. Die (light pink), diestrus; Pro (purple), proestrus; Female (red), mixed females; Male (blue), males (*n* = 6 datasets)

*Tests for data variability* We tested data variability *in each dataset* using two different methods. First, we evaluated equality of distribution shape between males and females using a two-sample Kolmogorov–Smirnov test after centering the variables to ignore mean differences. Second, we assessed difference in the variance between groups using Levene’s test for equality of variances. To compare variance *across datasets*, we first calculated coefficient of variation (CV) for each group in each dataset by dividing the standard deviation by the mean. We then compared the CV for each group across tests using one-way ANOVA (Analysis 1) or a Welch two-sample *T*-test (Analysis 2; Fig. 6). Statistical analyses and plots of these data were generated using R software.

## Results

### Behavioral analyses

We previously compared diestrus females, proestrus females, and males across three different tests for anxiety-related behavior, including open field, light dark box, and elevated plus maze (Fig. 2A) [5]. Across all tests, diestrus females exhibited higher anxiety indices than proestrus females, while a sex difference was found between diestrus and male groups only (Fig. 2A) [5]. Specifically, in the open field, there was a significant effect of group on the time spent in the center [*F*(2,39) = 5.93, *P* = 0.006], with post hoc test showing diestrus females spending less time in the center compared to both proestrus females (*P* = 0.006) and males (*P* = 0.044, Fig. 2A) [5]. In the light dark box test, we found a significant difference between groups in the time spent in the light compartment [*F*(2,37) = 21.63, *P* < 0.001], which was driven by diestrus females spending less time in the light than both proestrus females (*P* < 0.001) and males (*P* < 0.001, Fig. 2A) [5]. Finally, in the elevated plus maze test, we saw a significant effect of group on the time spent in the open arms of the maze [*F*(2, 37) = 5.33, *P* = 0.009], with diestrus females spending less time in the open arms compared to proestrus females (*P* = 0.008) and there was a similar trend in the diestrus–male comparison (*P* = 0.084, Fig. 2A) [5].

Notably, when the two female groups are merged and compared to males (Fig. 2A), none of the behavioral comparisons between males and females reached statistical significance including the open field test [*t*(30.06) = 1.05, *P* = 0.303], the light–dark box [*t*(26.08) = 1.75, *P* = 0.092], and the elevated plus maze [*t*(22.05) = 0.71, *P* = 0.483].

Taking the light–dark box test further as an example, we visualized the normal distributions of the data, comparing mixed female and male group distributions, as well as distributions of separate diestrus, proestrus, and male groups (Fig. 2B). We found a substantial overlap between males and mixed-females (76%), as previously reported for many neurobehavioral measures [35]. However, when females are separated by the estrous cycle stage, there is a high overlap between proestrus and males only (82%), but little overlap between males and diestrus (34%) and even less overlap within females, between proestrus and diestrus groups (20%) (Fig. 2B).

We then addressed data variability between both males and merged females, as well as between proestrus, diestrus, and males for all three anxiety tests. For the time spent in the center of the open field, we found equal variance between proestrus females, diestrus females, and males [*F*(2, 39) = 0.80, *P* = 0.456; Levene’s test]; we also found equal variance [*F*(1, 40) = 1.21, *P* = 0.279; Levene’s test] and equal distribution shapes (*D* = 0.2, *P* = 0.823; Kolmogorov–Smirnov test) between merged females and males. We then looked into the time spent in the light compartment of the light–dark box test and found equal variance between proestrus, diestrus, and males [*F*(2, 37) = 0.46, *P* = 0.633; Levene’s test]; we also found equal variance [*F*(1, 38) = 1.63, *P* = 0.209; Levene’s test] and equal distribution shapes (*D* = 0.25, *P* = 0.591; Kolmogorov–Smirnov test) between merged females and males. Finally, we examined the time spent in the open arms of the elevated plus maze and found equal variance between proestrus, diestrus, and males [*F*(2, 37) = 0.83, *P* = 0.443; Levene’s test]; we also found equal variance [*F*(1, 38) = 0.01, *P* = 0.925; Levene’s test] and equal distribution shapes (*D* = 0.14, *P* = 0.986; Kolmogorov–Smirnov test) between merged females and males.

Overall, these data show that including the estrous cycle stage as a variable allows us to find the sex difference in anxiety-related behavior, which would be masked if the mixed female group was compared to males. Interestingly, we also found that the significant effect of the estrous cycle was not accompanied by the increased female variability compared to males, for any of the measured outcomes. In fact, we see similar variability between male and female groups, whether taking into account the estrous cycle or not.

### Analysis of dendritic spine density

To extend our study to other neurobehavioral outcomes, we performed similar analyses of dendritic spine density in the ventral hippocampus (Fig. 3). We previously analyzed spine density in males, proestrus females, and diestrus females and found a significant group effect [*F*(2, 597) = 1907, *P* < 0.001], with proestrus females having a higher density than both diestrus females (*P* < 0.001) and males (*P* < 0.001; Fig. 3A) [5]. In this example, females either have significantly higher, or equal, dendritic spine density in comparison to males depending on their estrous cycle stage. Importantly, when the two female groups are merged, this dynamism in the sex difference is lost and merged females are observed to have higher spine density than males [*t*(540.91) = 16.04, *P* < 0.001; Fig. 3A].

When we analyzed this data using normal distributions (Fig. 3B), we found a partial overlap between males and mixed females (38%). However, after separating females by their estrous cycle stage, we found a large overlap between males and diestrus females (90%), and virtually no overlap between males and proestrus females (1%) or within females, between proestrus and diestrus (1%), illustrating how the information about the estrous cycle gives new insight into the data.

We also tested data variability for dendritic spine density in the ventral hippocampus. We found unequal variance between males, proestrus females, and diestrus females [*F*(2, 597) = 5.65, *P* = 0.004; Levene’s test], as well as between males and merged female groups [*F*(1, 598) = 530.88, *P* < 0.001; Levene’s test]. We also found that distribution shapes were unequal between merged females and males (*D* = 0.49, *P* < 0.001).

Overall, this data provides an example where females have higher variability than males, and a sex difference can be found without accounting for the estrous cycle. However, having the information about the estrous cycle explains where the sex-based variability is coming from and allows for a mechanistic insight, which is that the sex difference is driven by sex hormone changes in females.

### Gene expression analysis

We further looked into our molecular data, including ventral hippocampal gene expression of two genes: *Ptprt*, (encoding protein tyrosine phosphatase receptor type T), involved in the development of dendrite spines [36]; and *Htr2b* (encoding serotonin receptor 2b), important for anxiety-related behavior [37] (Fig. 4).

For *Ptprt*, we observed a similar pattern that we observed with the dendritic spine density data (Fig. 3). Comparing diestrus, proestrus, and males, we found a significant effect of group on *Ptprt* expression [*F*(2, 21) = 483.2, *P* < 0.001], with proestrus females having higher expression than both diestrus females (*P* < 0.001) and males (*P* < 0.001), and with males having higher expression than diestrus females (*P* = 0.002; Fig. 4A) [5]. When the two female groups are merged, this dynamic sex difference is reduced to the merged female group exhibiting higher overall *Ptprt* expression compared to males [*t*(16.18) = 2.72, *P* = 0.015; Fig. 4A]. We also created distributions for this dataset (Fig. 4B), and found that there is a small overlap between males and mixed females (23%), with the distribution of mixed females appearing notably flatter. After separating the female groups, there is a modest overlap between diestrus and males (35%), and there is no overlap between proestrus and males (0%) or within females, between proestrus and diestrus (0%), indicating these groups form entirely distinct populations in measures of ventral hippocampal *Ptprt* expression.

For the second gene, *Htr2b*, we found a significant difference between diestrus females, proestrus females, and males [*F*(2, 21) = 12.87, *P* < 0.001], with diestrus females having higher expression than both proestrus (*P* < 0.001) and male (*P* = 0.013) groups (Fig. 4A) [5]. When the two female groups were merged, however, we found no difference between males and females [t(21.61) = 0.65, *P* = 0.520; Fig. 4A].

We then looked into data variability, both among proestrus, diestrus, and male groups, as well as between the merged female and male groups. For expression of *Ptprt* in the ventral hippocampus, we found equal variance between proestrus females, diestrus females, and males [*F*(2, 21) = 0.19, *P* = 0.829; Levene’s test]; however, variance was unequal between merged females and males [*F*(1, 22) = 269.3, *P* < 0.001; Levene’s test], while distribution shape between these two groups were equal (*D* = 0.5, *P* = 0.126; Kolmogorov–Smirnov test). For expression of *Htr2b* in the ventral hippocampus, we found equal variance between proestrus, diestrus, and male groups [*F*(2, 21) = 1.96, *P* = 0.166; Levene’s test]; we also found equal variance [*F*(1, 22) = 3.99, *P* = 0.058; Levene’s test] and equal distribution shape (*D* = 0.38, *P* = 0.424; Kolmogorov–Smirnov test) between merged females and males.

In summary, we found *Ptprt* expression to follow the same pattern that we see with the structural dendritic spine phenotype; we found more variability in females than in males and that the sex difference, detectable when females are merged, is actually driven by the estrous cycle stage. With *Htr2b* expression, we see the pattern that we observed with anxiety-related behavior; males and females show similar variability and sex difference can only be detected when there is information about the estrous cycle stage.

### Analysis of the 3D genome interactions

Finally, we explore our previously published data derived from the unbiased chromosome conformation (Hi-C) assay (Fig. 5) [34]. This assay detects 3D genome interactions throughout the genome, and here we focus on CTCF loops (Fig. 1E), which allow long-range interactions between distant genomic regions, important for higher-order chromatin organization and gene regulation [38]. We explored these chromatin loops in sorted ventral hippocampal neurons and made the following comparisons: diestrus vs. male groups, as well as merged female (diestrus + proestrus) vs. male groups (Fig. 5). Importantly, we found an increased ability (1.65 times) to call sex-specific loops when comparing diestrus to males (260 differential loops), as opposed to comparing mixed females to males (158 differential loops; Fig. 5A) [34].

To illustrate this with an example, we present a loop involving *Adcyap1* (Fig. 5B) [34], an important stress- and estrogen-sensitive gene implicated in anxiety-related behavior [39, 40]. This 2-Mb loop is stronger in proestrus and males than in diestrus (Fig. 5B), and this is associated with differential *Adcyap1* expression among the three groups (Fig. 5C). Interestingly, this differential loop is also found in the mixed-female to male comparison (Fig. 5D) [34], further showing that the sex-specific dynamism that we observed, with proestrus becoming more similar to male *Adcyap1* in terms of gene looping and gene expression, is only detectable if we monitor the estrous cycle stage.

Overall, this data indicates that accounting for the estrous cycle stage in females helps identify sex differences in chromatin looping of relevance to chromatin organization and gene expression.

### Data variability across neurobehavioral measures

Finally, we decided to test our data variability across all neurobehavioral measures—behavior, hippocampal dendritic spine density, and gene expression—using the coefficient of variation (CV = standard deviation/mean), as a measure of relative variability, previously described in the meta-analyses performed by Prendergast et al. [22] and Becker et al. [21] (Fig. 6). When we calculated and compared the CV value for each group across the 6 datasets described here, we found no difference in variability between females and males whether females were separated by estrous cycle stage [*F*(2, 15) = 0.514, *P* = 0.608, Fig. 6A], or merged into one female group [*t*(9.94) = 1.87, *P* = 0.092; Fig. 6B].

In sum, our data are consistent with the data previously reported in mice and rats that females are, on average, not more variable than males [21, 22, 26]. However, our data also clearly show that this finding is not, at all, predictive of whether the estrous cycle plays an important role in regulating the outcome of interest.

## Discussion

In this manuscript, we show that accounting for the estrous cycle significantly increases the resolution of the neuroscience studies and allows for: (a) identification of masked sex differences; (b) mechanistic insight(s) into the identified sex differences, across different neurobehavioral outcomes, from behavior to molecular phenotypes. We strongly encourage the neuroscience community to incorporate the estrous cycle information in their study design and data analysis, whenever possible, and we debunk some myths that tend to de-emphasize the importance and discourage the inclusion of this critically important biological variable (Table 1).

**Table 1:** Misconceptions regarding the estrous cycle

1. “Females are *not* more variable than males” means that the estrous cycle does not have an effect on the outcome of interest	This is an often used argument to rule out the importance of the estrous cycle’s effect on the outcome of interest. We show here that female variability is not predictive of the effect of the estrous cycle on variables from behavior to molecular phenotypes. Unless the study includes the information about the estrous/menstrual cycle stage or hormone levels measurements, it should not make any conclusions about the effects of cycling ovarian hormones
2. The estrous cycle tracking is a stressor and may represent a hidden variable in the data if incorporated in the study	If performed properly by trained individuals, tracking of the estrous cycle is not stressful to rodents. If researchers are concerned with more handling of females than of males, they can always handle males in parallel with the estrous cycle tracking. In any case, not having the information about the hormonal state of the animals is much more of a hidden variable than the stress imposed by vaginal smearing
3. The studies should start with smaller cohorts of mixed male and female animals and then proceed with bigger follow-up studies if any sex-biased “trends” in the data are observed	The under-powered studies can be misleading and even lead to further exclusion of female animals from the studies. As shown here, many times sex difference can be masked if the estrous cycle is not accounted for, and this is especially true when smaller numbers of animals are used. We warn that this practice may lead to inaccurate interpretation of lack of the influence of sex on the outcome of interest, followed by the use of male animals only
4. Monitoring the estrous cycle requires an expert in reproductive endocrinology	While systematic tracking and staging of female animals can be labor-intensive, the skills required are simple in comparison to the average techniques used in neuroscience, and the increased resolution of the data are worth the effort. Several resources are available to assist laboratories unfamiliar with the procedure (see Practical consideration and recommendations section). Further, if systematic tracking is not feasible, taking single smears at the time of data collection can be done quickly and cheaply with minimal training and can provide valuable information for the field

Since the NIH and other funding agencies mandated the use of both sexes in grant proposals, there are an increasing number of studies that include both males and females, as opposed to the past preclinical neuroscience research that largely focused on males [19]. The SABV policy was certainly a critical step towards more equitable research practice and has sent an important signal that male-only research is not scientifically and socially responsible, since it does not apply to women and people across genders. However, since the policy is not very strict and there is no system in place to keep researchers accountable, there has been a varied response to this policy after funding is awarded. Some researchers still focus on males; others use mixed male and female groups that are typically underpowered to detect sex differences; a smaller percentage of researchers actually run studies that are designed to test the effect of sex [29, 30]. This varied response and its outcomes also raised concerns that sex differences are now misreported and misinterpreted [32], and that this practice can hurt the precision medicine initiative in the long-run [33].

While we acknowledge that some researchers do not have sex difference as a focus of their research, the moment males and females are being included, the effect of sex should be assessed by designing a well-powered study to incorporate sex as a variable in data analysis. First, considering the sex differences already identified across diverse neurobehavioral outcomes, one cannot reasonably suggest that sex will not affect a particular measure without testing this assumption. Identification of such sex differences is particularly important for understanding the understudied female brain, since it contextualizes previous male-specific findings. Second, the focus of preclinical neuroscience research should reflect the need to understand the near-ubiquitous sex bias in prevalence and symptomatology of brain disorders. For instance, each study of depression- and anxiety disorder-related phenomena should address the important question of why women are at twice the risk for these disorders than men are. In this case, finding female-specific mechanisms and treatments could be transformative for two thirds of the patient population. Third, running small, underpowered cohorts of mixed male and female animals risks missing an existing sex difference which can lead to exclusion of females in future experiments, as some researchers will continue with only males if no sex difference is found initially (Table 1). This can further harm the understudied field of women’s health research.

Considering the importance of sex difference research for women’s health, and the challenges faced by this field, then, one might wonder whether emphasizing the need to study the estrous cycle represents just another roadblock to equitable research. In fact, this variable was the major reason why females were excluded from neuroscience studies for decades [19]. In response, there was a concerted effort within the community to show that the variability of females is not higher than that of males [21, 22, 26] so that more people feel comfortable including females, avoiding expensive studies that may incorporate multiple female groups to account for varying sex hormones. So, would calls for incorporating the estrous cycle now bring us backwards?

In fact, here we want to show why “bringing back” the estrous cycle variable to the main stage is important and can help us improve the health of women and make research more gender-inclusive, while also enhancing the ability to detect and interpret sex differences. First, we show that in our anxiety-related behavioral data and gene expression results for *Htr2b,* a gene implicated in these behaviors, we can find sex differences only if we account for the estrous cycle stage. This is important because it was suggested that the old tests that were developed for males, such as the elevated plus maze, may not be applicable to females as they cannot re-create increased female risk for anxiety disorders reported in humans [23, 41]. What we demonstrate, though, is that proper classification of females will show the same trend that we see in humans, which is that low-estrogenic female mice (diestrus) show higher anxiety indices than high-estrogenic females (proestrus) and males, thus recreating the fact that sex hormone withdrawal in humans is a trigger for increased anxiety and depression symptoms [8, 9] or other reproduction-related disorders such as PMDD [10], postpartum depression [11, 12], and perimenopausal depression [8, 14]. At the structural level, this vulnerability is seen as a drop in dendritic spines in the mouse ventral hippocampus following estrogen withdrawal; a similar finding is also reported in humans as reduced hippocampal gray matter following an estrogen drop [2, 42]. Thus, we would completely miss this clinically relevant finding if the estrous cycle information was not available. For behavior, no sex difference would be reported which could lead to further exclusion of females. For dendritic spines, the mechanism for the observed sex difference would be left unknown.

This important effect of the estrous cycle is not only applicable to behavioral, structural, and candidate gene expression data, but we also observe it in cutting-edge epigenomics data. The neuroepigenomics field [43] has been largely focused on the male brain, and the chromatin dynamism that we see across the estrous cycle, in terms of chromatin accessibility [5] and 3D genome organization [34], is critical for understanding the female-specific gene regulation that contributes to both brain physiology and disease risk. As shown in the example here, we can identify many more sex-specific loops when we account for the estrous cycle. Further, these dynamic changes in gene regulation are likely to underlie female-specific vulnerability to brain disorders and will offer new opportunities for treatment [17].

We want to emphasize that the increased resolution that we are seeing in our studies is because the hormonal status is a sex-specific factor that is more precise than sex. Multiple researchers have indicated that sex is a complex, multi-layered variable that should be used as a proxy rather than a variable that explains sex-based variation [35, 44]. We were warned that male and female populations are largely overlapping in both animal [35] and clinical [45] research and that over-interpreting sex differences can bring us further from truth both in experimental research and in medicine [33, 35], and it hurts transgender and gender-nonconforming individuals [31]. With our population graphs, we show that incorporating the estrous cycle stage, an exemplary sex-specific variable, increases our resolution so we can separate different populations and better interpret our data. The ovarian cycle stage is also gender-independent, so our findings are applicable to all individuals who experience ovarian hormone fluctuations, across gender, including cis women, non-binary individuals, and transgender men who menstruate.

Our results also debunk the myth that female variability is predictive of the importance of the estrous cycle for the regulation of the outcome of interest. This frequently touted misconception states that it is now proven that the estrous and menstrual cycles do not make females more variable than males, and that this means that (a) females *deserve* to be studied and that (b) ovarian cycles are not important experimental considerations [24, 46, 47]. First, we show that it is possible to not find any difference in the variability between males and females, and still find an effect of the estrous cycle. In fact, our behavioral data show no sex difference in variability; but, importantly, in the same datasets, sex differences will not be detected unless the estrous cycle information is incorporated. Therefore, female variability should not be used as a proxy for the effects of sex hormones on the brain and behavior (Table 1). We discourage researchers from perpetuating this misconception because it hurts the research aimed at understanding these effects of ovarian hormone fluctuations to help the health of women and other menstruating individuals.

As mentioned previously, sex hormone fluctuation is one of the most important indices that determines women’s health outcomes and, in particular, there is no other factor, except for possibly trauma, that may acutely increase the risk for mental disorders as can a drastic shift in systemic ovarian hormone concentration. We see this postpartum, premenstrually, and at perimenopause. Yet, stress or trauma is one of the most studied factors in mainstream neuroscience and psychiatric research, while we are far behind with studying the effects of ovarian hormone changes. And, it is difficult not to assume that the main reason for this is that we are talking about a female-specific factor. We hope that our colleagues will recognize that “women’s health” should not be a niche field but that, rather, it is the health of half of the population, 50% of whom are in the reproductive period and dealing with the ovarian cycle. As such, no further information is needed to justify why females *deserve* to be studied. The time is now to improve our understanding of the female brain and the health of women and other menstruators, and for this, we need to acknowledge the ovarian cycle as a critical biological variable that shapes the brain and behavior.

### Practical consideration and recommendations

Here we provide evidence that incorporating the estrous cycle information makes the data across neuroscience studies more interpretable and increases our ability to discover and explain sex differences. Thus, we recommend that neuroscience researchers utilizing reproductive-aged female animals incorporate the estrous cycle information whenever possible. This recommendation certainly raises concerns regarding the practicality and feasibility of this approach, particularly for the studies that incorporate multiple treatment groups and large numbers of animals. So, we would like to address this issue here.

Ideally, the estrous cycle should be tracked systematically, by daily vaginal smearing for three consecutive cycles, approximately for 2 weeks, so that the experiments can be performed with properly staged female animals [5, 34]. These experiments give the best information about the estrous cycle’s effect on the outcome of interest since we can predict and maximize the number of animals in each group, and minimize staging errors. Notably, in our studies, we have focused on two extreme phases of the cycle, proestrus (high estrogen, low progesterone) and early diestrus (low estrogen, high progesterone), which mimic human follicular and luteal phases, respectively (Fig. 1A). This approach gives us optimal information about the effects of hormonal fluctuations on the brain and behavior; it is translationally relevant, and requires the use of a reasonable number of animals while maximizing data quality. This approach, or an approach where all four stages of the cycle are included, should be used whenever the effect of the estrous cycle is the central question of the study, or when the estrous cycle has already been demonstrated to affect an outcome of interest.

However, we acknowledge that this approach is more difficult to implement in the studies that incorporate multiple treatment groups and large numbers of animals. In that case, we suggest an approach that is less labor-intensive and more typically used in practice, which is taking vaginal smears at time of data collection, either after the test was performed (e.g., behavior) or postmortem (for histology and molecular analyses). Even if these studies are underpowered to find differences between females at specific estrous cycle stages, merging stages with similar hormonal profiles can still give insights into whether the outcome of interest is affected by ovarian hormone fluctuations. As an example, in our study of the combined effect of early life stress and adolescent stress, we had four treatment groups with females and males in each group and, thus, systematic tracking of the cycle was not possible [48]. However, the stress-induced effect was seen in high-estrogenic females only (the combined group of late diestrus, proestrus, and early estrus females), so having the information about the estrous cycle stage at the time of testing allowed us to conclude that early life stress disrupts the protective role of estrogen on anxiety-related phenotypes in females [48]. This mechanistic insight into the sex-specific effect of stress on behavior would not be possible without having the cycle information.

However, we would like to warn the researchers that some manipulations (e.g., cocaine or stress exposure) may disrupt the regularity of the rodent cycle and that careful examination of the estrous cycle patterns across the experimental groups is always advised in order to spot possible biases. As an example, animals may appear to be overwhelmingly in a single cycle stage, such as estrus or diestrus, in which case the estrous cycle and its effects need to be re-examined with a more comprehensive approach.

Further, for transcriptomics and epigenomics analyses with budgets limited to one male and one female group, the researchers may decide to control for the effect of the cycle by selecting females equally distributed across the cycle or in one stage only (e.g., low- or high-estrogenic phase), based on information acquired using vaginal smears post-mortem. In general, we believe that the information of the estrous cycle stage of the animals should be provided in each study, together with other major animal information such as strain, age, sex, light–dark cycle, housing conditions, time of testing, etc. Even if this information is not directly analyzed by the researchers, it represents valuable information for the field that can inform future analyses.

Finally, as we consider the estrous cycle tracking, we would like to highlight the two biggest misconceptions related to this procedure that make researchers reluctant to include it in their studies: (1) that estrous cycle determination requires expertise in reproductive endocrinology; (2) that this procedure is a major stressor to the animals (see Table 1). In fact, vaginal smears are easy to perform and, once the person is well-trained and practiced, the stress imposed on the animal is minimal. The procedure is also inexpensive and there are multiple resources that researchers can use as guides for monitoring the rodent estrous cycle [5, 49–52].

As the field embraces the importance of this variable, we hope that we may see development of the approaches that will allow for even easier estrous cycle tracking, with minimal manual labor, perhaps by devices that will allow continuous tracking of the variables that can accurately predict the estrous cycle stage such as temperature, sleep pattern, food intake, and activity levels. We believe that there is no reason for a sophisticated field such as neuroscience, which regularly uses light-driven genetic tools and mini-scopes, to reject the inclusion of the estrous cycle tracking out of inconvenience.

### Perspectives and significance

Here we provided experimental evidence that the estrous cycle information increases the resolution of the preclinical neuroscience studies while critically informing women’s health research and allowing for more gender-inclusive research practices. We encourage all researchers to consider implementing the estrous cycle tracking in their study design, provide some practical considerations and recommendations, but also envision future technological innovations that will facilitate this process, as long as there is desire and need to study this important variable.

## Data Availability

All of the data re-analyzed in this manuscript are available at the following Figshare link: https://doi.org/10.6084/m9.figshare.20301282.v3
